# Recent Hydrophobic Metal-Organic Frameworks and Their Applications

**DOI:** 10.3390/ma11112250

**Published:** 2018-11-12

**Authors:** Ruth Antwi-Baah, Heyang Liu

**Affiliations:** 1 School of Biological and Chemical Engineering, Zhejiang University of Science and Technology, Hangzhou 310023, China; maadede97@gmail.com; 2 Zhejiang Provincial Key Lab for Chem. & Bio. Processing Technology of Farm Product, Hangzhou 310023, China

**Keywords:** metal-organic framework, hydrophobicity, adsorptive separation, gas separation and storage, self-cleaning, liquid marbles

## Abstract

The focus of discussion of this review is the application of the most recent synthesized hydrophobic metal-organic frameworks (MOFs). The most promising hydrophobic MOFs are mentioned with their applications and discussed. The various MOFs considered are sub-sectioned into the main application areas, namely alcohol adsorption and oil/water-alcohol/water separation, gas separation and storage, and other applications such as self-cleaning and liquid marbles. Again, the methods of synthesis are briefly described, showing how the features of the end product aid in their applications. The efficiency of the MOF materials and synthesis methods are highlighted and briefly discussed. Lastly, the summary and outlook section concludes the write-up giving suggestions that would be useful to present-day researchers.

## 1. Introduction

Metal-organic frameworks (MOFs) are an attractive crystalline class of porous materials that entail metal ions and organic bridging ligands coordinated together in a controlled manner to enhance several requisite features over their conventional porous inorganic and organic counterparts such as activated carbons and zeolites [1,2,3,4,5]. These materials have gained enormous attention from various scientists around the world.

Over the years a vast variety of MOFs have been produced and have served diverse purposes in a wide range of industries. MOFs have attracted the attention of many scientists due to their large surface area, designable pore structure and functionality, high thermal stability and huge porosity [1,6,7]. These special properties have made them promising in diverse applications including gas storage [8,9] and separation [10,11,12,13], chemical sensing [14,15], catalysis [16,17,18,19,20], wastewater treatment [21], proton conductors [22], and drug delivery [23]. Down the line, a disturbing problem was realized with these impactful materials upon their application in moisture-bound areas; most MOFs degrade under moisture conditions, limiting their functions in some industries [6,24].

Instability of MOFs is ascertained when the original structure of a MOF is distorted, and this is mainly realized by performing powder X-ray diffraction (XRD) and nitrogen sorption isotherms. With the sorption isotherms, the surface area of the MOF reduces and, with the XRD, the pattern of the peaks metamorphose or change [24].

It was reported that MOFs get degraded via two main mechanisms namely: (1) ligand displacement and (2) hydrolysis [25]. These mechanisms were arrived at through computational chemistry and confirmed experimentally. The ligand displacement occurs when a water molecule gets trapped into the metal-ligand M–O bond of the framework leading to the formation of a hydrated cation and the release of a free ligand:

(1) $$M^{n+}-L^{n-}+H_{2}O\to M^{n+}-(OH_{2})\cdots L^{n-}$$

On the other hand, in hydrolysis reaction, the metal-ligand bond is broken, and water dissociates to form a hydroxylated cation and free protonated ligand:

(2) $$M^{n+}-L^{n-}+H_{2}O\to M^{n+}-{\left(OH\right)}^{-}+{HL}^{(n-1)-}$$

Conversely, hydrophobic MOFs possess water-resistant sites on their surfaces that curb this situation.

In the quest to curb this situation, another breed of MOFs known as hydrophobic MOFs have been synthesized by several scientists around the world. These MOFs have either highly strong metal-ligand bonds (which are difficult to hydrolyze) or contain organic struts that bear hydrophobic groups [26,27,28,29,30,31]. As the name implies, these are simply MOFs that repel water, thus, inhibiting the penetration of water molecules into their pores [26]. Hydrophobicity of MOFs is mainly classified by their contact angles with water. MOFs with contact angles (CAs) between 90° and 150° are referred to as hydrophobic, those with CAs greater than 150° are termed superhydrophobic [30] and those with CAs much more above this figure are termed ultrahydrophobic [32].

In the past years, several hydrophobic MOFs were synthesized to solve the water-instability problem. The Cohen group, through postsynthetic modification (PSM), introduced hydrophobic alkyl chains on a series of Isoreticular metal-organic frameworks (IRMOFs) and MIL-53(Al)-NH_2_, rendering them hydrophobic and superhydrophobic respectively [30]. Another group complemented this work by directly synthesizing a series of highly hydrophobic Cu-based MOFs with novel perfluorinated aromatic linkers [31]. Kitagawa and coworkers devised an external-surface-corrugated superhydrophobic porous coordination polymer (PCP) with the use of aromatic hydrocarbon building unit [27]. Onwards, quite several hydrophobic MOFs, which are derivatives of some of the first works have been made by different researchers. Recently, a novel MOF called *o*CB-MOF-1 whose crystal surface can be switched between hydrophobicity and superhydrophilicity by chemical treatment was reported [33]. Such switchable porous materials could be useful for obtaining smart porous surfaces such as membranes and coatings and serve a vast pool of purposes. Despite this, the direct application of many of such MOFs remains uninvestigated.

In addition, it is important to note that a group of scientists has recently produced a new method of activating open coordination sites (OCS) of MOFs, with the use of inert chloromethanes [34]. It is common knowledge that activation, that is, the removal of solvent molecules coordinated at the OCSs is a very essential step prior to the use of MOFs. The chemical activation method involving dichloromethane (DCM) [35] or trichloromethane (TCM) [36] was found to be a promising alternative to the conventional thermal activation (TA) method whereby the structural integrity of the framework is unpreserved in some cases [37,38,39].

**Figure 1:**
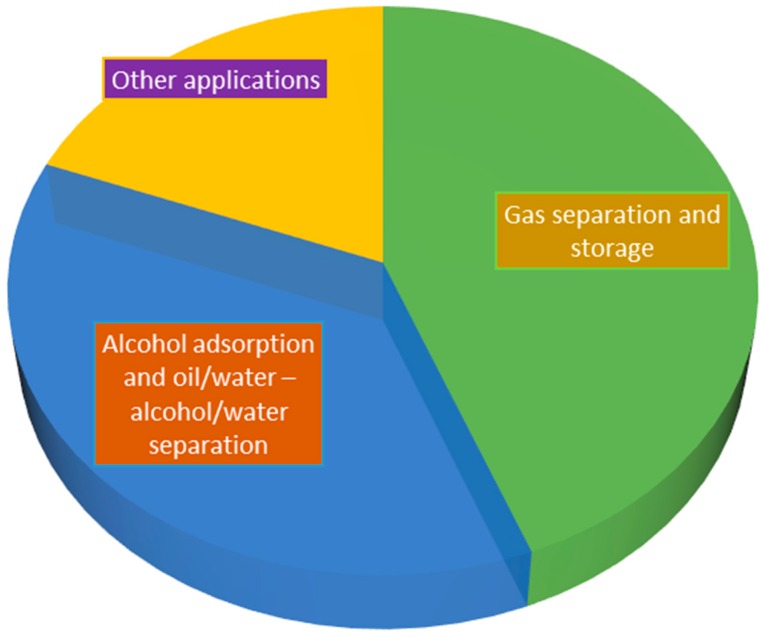
Pie chart describing the applications of hydrophobic MOFs presently.

In this review, we briefly present how some of the most recent hydrophobic MOFs by the manipulation of these two broad classes namely: (1) direct synthesis and (2) postsynthetic modification (PSM) were made, and their applications. The strategies and chemistry of postsynthetic modification will not be discussed here because it has been extensively discussed by some writers [40,41,42,43]. This rather short review is not intended to be all-inclusive in the entire body of published literature. Instead, we intend to mention several hydrophobic MOFs that have been employed in some application areas so far. We seek to bring to light the properties that these MOFs possess that make them function as hydrophobic particles, and hence their respective applications. Figure 1 shows the various applications discussed in the literature, thus, alcohol adsorption and oil/water-alcohol/water separation; gas separation and storage; and other applications such as self-cleaning and liquid marbles. Moreover, a concise description of the synthesis of these materials is also given. After the introduction, we highlight the applications of hydrophobic MOFs section by section, indicating the important criteria for each application area. Lastly, we shortly present our view of the setbacks in this field of research and the possible way forward.

## 2. Alcohol Adsorption and Oil/Water-Alcohol/Water Separation

The development of renewable and clean energy sources such as bioalcohols has attracted attention in recent years because of emerging scarcity of fossil resources and the accompanying critical environmental concerns [44]. Adsorptive separation by using porous materials is considered to be one of the most cost-effective and environmentally friendly methods of recovering bioalcohols from fermentation broth produced from biomass. The use of hydrophobic MOFs for adsorptive separation of alcohol/water mixture is a probable solution to this problem as they have a less strong affinity to water compared to other porous materials such as activated carbons, zeolites and polymeric resins which have as well been tested for this application [45,46,47,48,49]. Adjustable internal surface properties and tunable pore size [50,51], and hydrophobicity [45,52] are important criteria for the adsorption performances of such materials.

Moreover, hydrophobic MOFs for this same common reason of moisture stability hold promise for oil/water separation compared to other porous adsorbent materials such as organoclays, zeolites, activated carbons, sand and cotton fibers which have been used to tackle oil spillage [53,54,55]. Furthermore, fabricated functional materials are significant for preparing efficient oil-absorbing materials such as microporous polymers [45], macroporous gels [56], cross-linked polymer gels [57], meshes/membranes [58], sponges [59] and graphene [60]. This points out the necessity of the use of hydrophobic MOFs to solve such problems. Wettability of surface area, water resistance, and aqueous stability are the most important criteria for this application.

**Figure 2:**
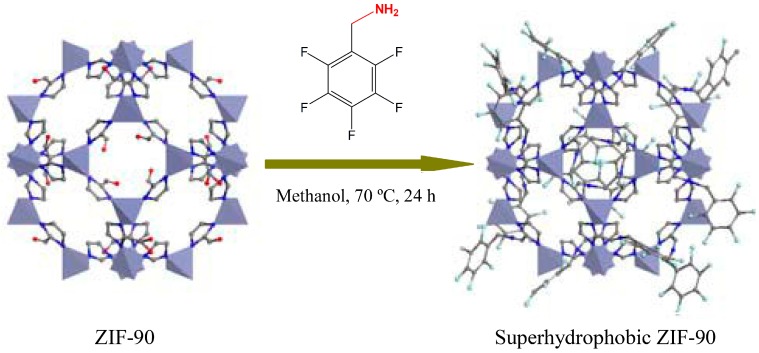
Scheme of the synthesis of superhydrophobic ZIF-90 via an amine condensation reaction. Reprinted with permission [63] Copyright 2016, Royal Society of Chemistry.

**Figure 3:**
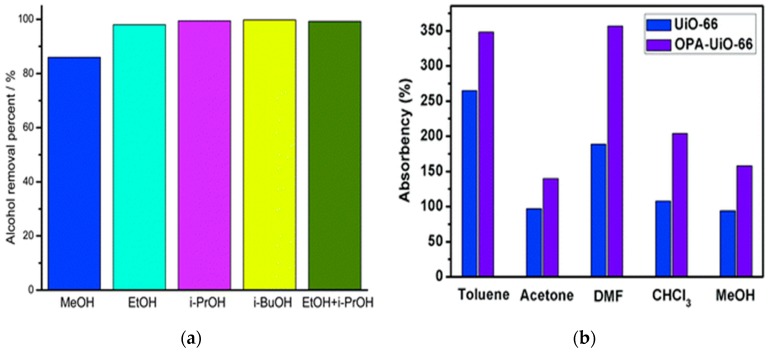
(**a**) Adsorptive separation performance of the superhydrophobic ZIF-90 for removal of alcohols from alcohols/water mixtures Reprinted with permission [63] (copyright 2016, Royal Society of Chemistry); (**b**) Absorption of organic solvents with UiO-66 before and after OPA modification. Reprinted with permission [62] Copyright 2017, Royal Society of Chemistry.

The exterior of hydrophobic surfaces and in many cases superhydrophobic surfaces have low surface energy [61,62] which is a phenomenon of superhydrophobic (SH) MOFs. Alcohol adsorption from aqueous solution is one essential application area for such materials. Liu et al. fluorinated Zeolitic Imidazolate Framework, ZIF-90 through an amine condensation reaction with pentafluorobenzylamine [63]. This type of post-functionalization was possible due to the presence of aldehyde groups in the ZIF-90 framework (Figure 2), proved by X-ray photoelectron spectroscopy (XPS) and Fourier transform infrared (FT-IR) spectra. XRD proved that the morphology of the as-prepared ZIF-90 was maintained after fluorination. The reported CA as shown in Table 1 was 152.4° which proved its hydrophobicity. It achieved a 98% recovery of ethanol from an ethanol/water mixture within 20 h of contact whereas the unfluorinated or unmodified ZIF-90 adsorbed only 7% of ethanol from a similar mixture. The superhydrophobic MOF exhibits high adsorptive separation qualities for the removal of other bioalcohols such as methanol, isopropanol and butanol as well as its mixtures (Figure 3a). Interestingly, the material can be regenerated by simple thermal regeneration under vacuum and is highly adsorptive even after five successive cycles. These qualities make this material suitable for recovery of bioalcohols from aqueous solution and can be employed commercially to produce biofuels.

Recently, the Burg group presented a new method called nanomechanical mass correlation spectroscopy (MCS) [67] to measure the effective mass density of MOF nanoparticles in different solvent systems [68]. Three different MIL-101(Cr) MOF species were employed: MIL-101(Cr) nanoparticles and its derivatives functionalized at the coordinatively unsaturated metal sites with pyridine or pyrazine using postsynthetic modification [69]. MIL-101(Cr) with different inner pore functionalizations were suspended in binary mixtures of ethanol and water, and methoxyperfluorobutane (HFE-7100) and ethanol. Mass fluctuations resulting from the flow of this suspension through a suspended microchannel resonator were measured. The pyridine-modified MOF, which is more hydrophobic than the other two kinds, records a lower effective density in the polar mixture, which is probably due to increased ethanol content within the particles. It was said that this occurred because the particles aggregated with ethanol in the interstices and/or ethanol solvation layer was formed around the nanoparticles. The tuning of the inner functionalization of the MOF is also a contributing factor to this effect. The differences observed contribute new information about the specific interaction between the different solvent components and the internal surface. Again, the findings in this work show new opportunities for the use of MOF nanoparticles in the separation of solvent mixtures founded on the selective enhancement of a solvent component in the pores.

**Figure 4:**
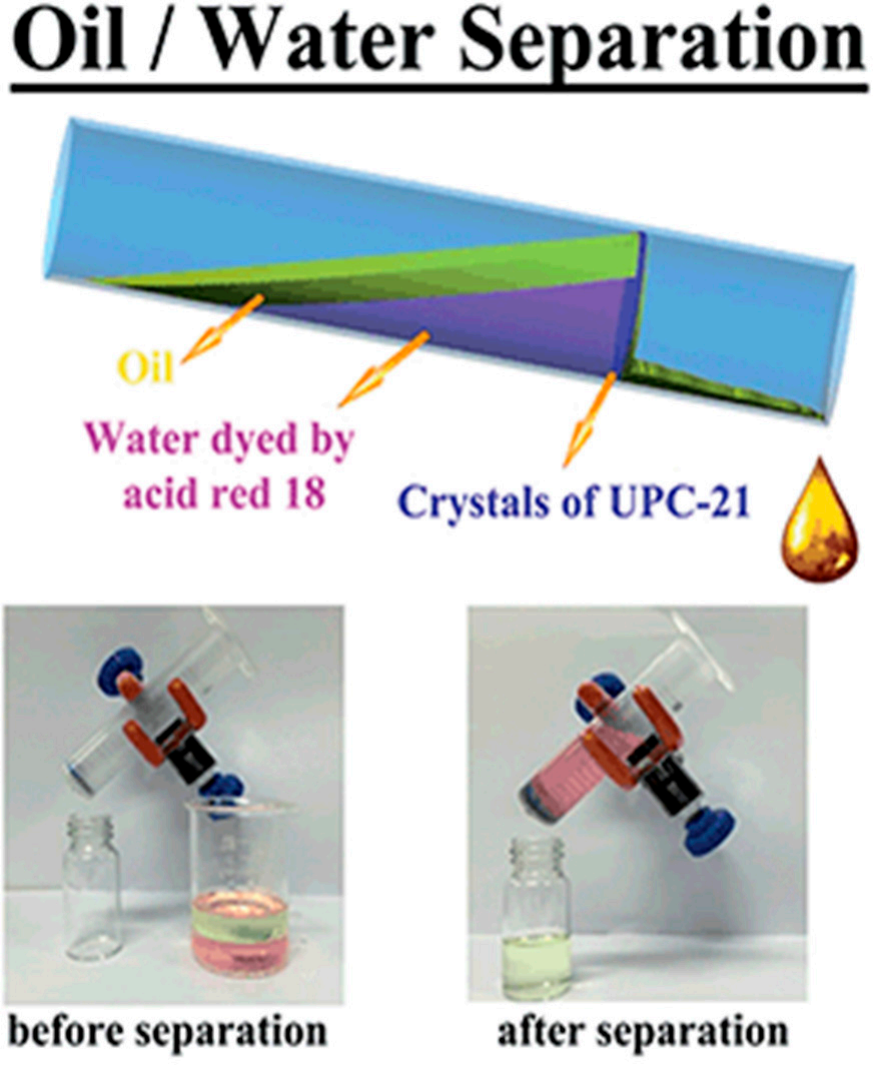
Photographs showing the status of separation experiments, oil/water separation efficiency. Water was colored with acid red 18 for observation. Reprinted with permission [65] Copyright 2016, Royal Society of Chemistry.

Oil/water and alcohol/water separation is another prime use of hydrophobic MOFs. Zhang et al. conducted a diauxic growth strategy on their previous MOF UPC-21 making it highly hydrophobic, with CA 145 ± 1° and exhibiting high oleophilicity [65]. The filtrate obtained from the initial MOF synthesis with the ligand H_4_L and metal compound Cu(NO_3_)_2_ was further placed again in the same condition as the previous MOF to obtain highly hydrophobic UPC-21 with high yield. The main contributing factor to the hydrophobicity of this MOF was the intelligent choice of organic ligand H_4_L. It carries multi-aromatic hydrocarbon units in its pentiptycene core, which are hydrophobic and arranged in a 2D layer, and is the key reason for the hydrophobic behavior of UPC-21. It was used to separate water from several organic solvents and oils by simple filtration method and achieved approximately 99% separation efficiency. Figure 4 depicts how the separation was conducted. It is a promising material for oil recovery during the occurrence of oil spills.

**Figure 5:**
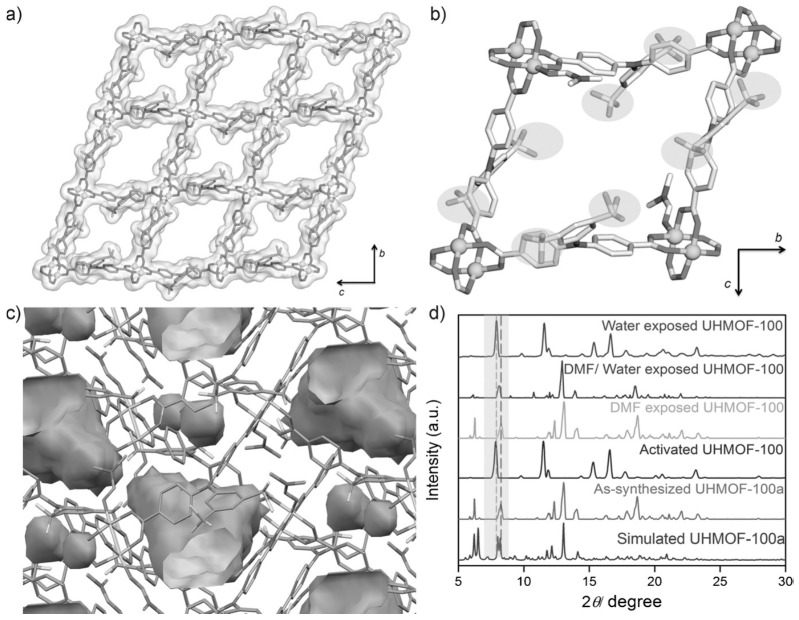
(**a**) Orthographic surface view of as-synthesized UHMOF-100 a; and (**b**) a single fluorous pores of UHMOF-100; (**c**) decorated fluorous voids on the Connolly surface; (**d**) PXRD profiles for different phases of UHMOF-100, validating its soft porous crystallinity and water stability. Reprinted with permission [32] Copyright 2016, Wiley—VCH.

**Scheme 1:**
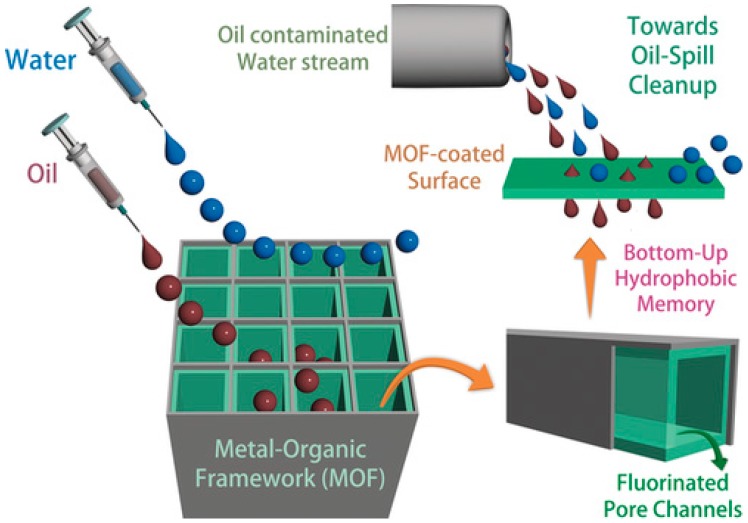
Schematic illustration of fluorinated linker-based predesigned pore surface corrugations exploited as a potential method to obtain ultrahydrophobicity in MOFs. Reprinted with permission [32] Copyright 2016, Wiley—VCH.

The organic linker component has an impactful influence over controlling the chemical and structural properties of the resultant material [3,70]. Hence, the systematic synthesis of new functionalized MOFs requires a meticulous choice of the organic linker component. In view of this, Mukherjee et al. synthesized an ultrahydrophobic MOF named UHMOF-100 with a careful choice of the organic ligand [32]. The new carboxylic low symmetry organic linker was cleverly synthesized, having in mind that such have a remarkable impact on the contact surface traits of the resulting material with liquid water [71]. The initial product formed, named UHMOF-100a contained dimethylformamide (DMF) guest molecules which were washed off to obtain UHMOF-100. Powder X-ray diffraction (PXRD) patterns show that the porous crystallinity and water stability of the latter are far higher than that of the former (Figure 5d). UHMOF-100 has by far recorded the highest water CA of approximately 176° and this is evident from Table 1. UHMOF-100-spray-coated polymeric hydrophobic membrane (UHMOF-100/PDMS/PP) was exploited in oil-water separation (Scheme 1), with substantial oil hexadecane, crude oil, toluene, biodiesel, and CCl_4_. The absorption capacities ranged from 40 wt.% to 70 wt.% with negligible difference in absorption capacities even after 10 cycles. Again, when water-in-oil emulsions prepared from dichloromethane, toluene and hexadecane were each poured on the upper side of UHMOF-100/PDMS/PP, emulsion droplets instantly demulsified. Oil components permeated right away through the membrane retaining water wholly above the membrane. It also proved efficient in alcohol-water separation by showing a sharp contrast between alcohol sorption isotherms and that of water. The performance of this MOF is partially attributed to unsaturated copper open metal sites and guest-accessible voids (Figure 5c).

**Scheme 2:**
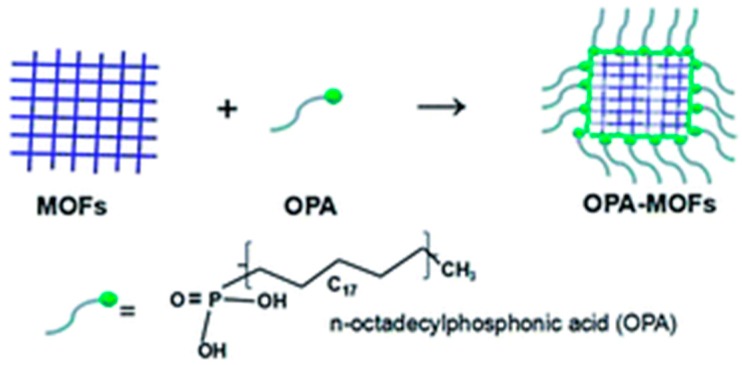
Molecular-level external modification for superhydrophobic MOFs using OPA. Reprinted with permission [62] Copyright 2017, Royal Society of Chemistry.

Modification of the external surface of zirconium-based MOFs via a facile method as shown in Scheme 2 through the incorporation of n-octadecylphosphonic acid (long alkyl chains) was performed by Ma et al. [62]. One important objective of this work was to impart hydrophobicity on the MOFs without the intact structure and internal channel environment being adjusted. It resulted in a superhydrophobic MOF of CA above 150°, was also used for oil-water and alcohol-water separation and absorbed up to about 99% of oil from oil-water mixture (Figure 3b).

Again, obtaining hydrophobic MOFs by coating the MOF surface with hydrophobic polymer crystals is inappropriate for this application. The reason is that these layers interact weakly with the water and do not promote water droplets from spreading on its surface [42]. The use of hydrophobic organic linkers in synthesis usually results in low surface area MOFs and is very evident in UHMOF-100 with BET of 469.2 m^2^**·**g^−1^. UHMOF-100 showed the highest efficiency for crude oil but the reverse was true for UPC-21 MOF. OPA-UiO-66 performed a better methanol adsorption than the fluorinated ZIF-90 MOF. Toluene performed much better with OPA-UiO-66 than UHMOF-100. The alcohol removal percentage with fluorinated ZIF-90 increased with a decrease in polarity of adsorbate. These interesting observations reiterate that the methods used for the synthesis of the hydrophobic MOFs were effective for their respective applications; varying materials and methods yield varying specific results upon application.

## 3. Gas Separation and Storage

Gas separation and storage has been one strong application area for MOFs as a whole. The metal clusters linked by organic ligands in MOFs yields porous three-dimensional networks with large pore volumes, high surface areas, low densities, good thermal stability, controlled porosities and the possibility of adjusting the pore size and chemical compositions [72,73,74,75]. These properties make this class of materials highly suitable for gas separation and storage [76,77,78,79]. Adsorption selectivity and uptake capacity are the most important for gas separation purposes [80]. The separation of various gases is an essential industrial process [81,82,83,84,85] because of the high quality and purity requirements for their practical use [86,87,88,89,90]. Again, carbon dioxide is a greenhouse gas that needs to be reduced in the atmosphere to avoid the dramatic consequences of global warming. Increased surface area, pore size, and moisture stability of MOFs are the most efficient approaches to maximize CO_2_ uptake [91,92]. Hydrophobic MOFs promise to be of highly valuable use in this rather broad area because of their structural stability in moist environments.

**Figure 6:**
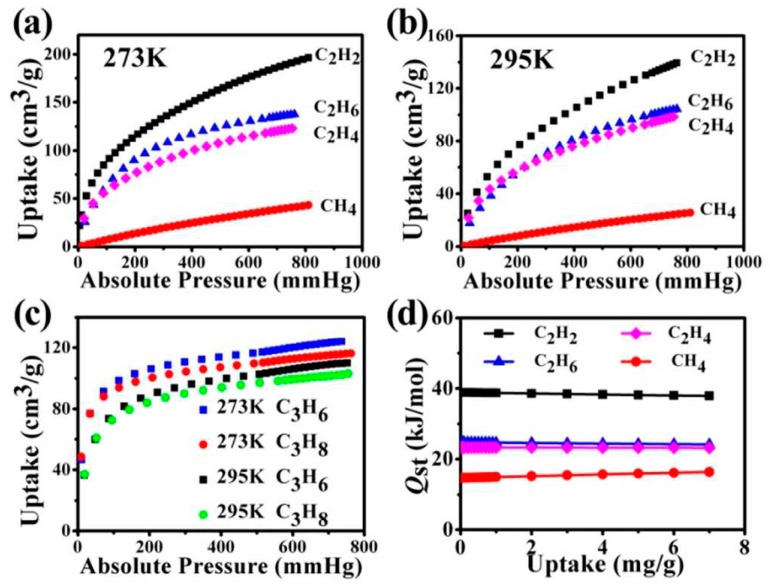
Hydrocarbon adsorption of UPC-21. The C_2_H_2_, C_2_H_4_, C_2_H_6_ and CH_4_ adsorption isotherms for UPC-21 at 273 K (**a**); and 295 K (**b**); (**c**) The C_3_H_6_ and C_3_H_8_ adsorption isotherms for UPC-21 at 273 K and 295 K; (**d**) The isosteric heat of adsorption for C_2_H_2_, C_2_H_4_, C_2_H_6_ and CH_4_ Reprinted with permission [65] Copyright 2016, Royal Society of Chemistry.

**Figure 7:**
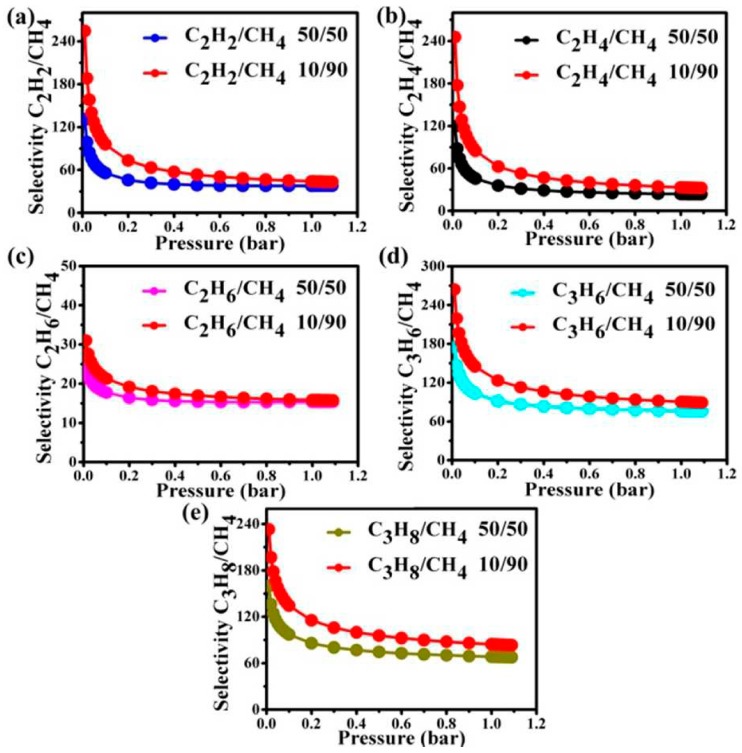
Selective adsorption of UPC-21. The calculations of the ideal adsorbed solution theory (IAST) selectivities for mixtures of C_2_H_2_/CH_4_ (**a**); C_2_H_4_/CH_4_ (**b**); C_2_H_6_/CH_4_ (**c**); C_3_H_6_/CH_4_ (**d**); C_3_H_8_/CH_4_ (**e**) at 295 K. Reprinted with permission [65] Copyright 2016, Royal Society of Chemistry.

The UPC-21 hydrophobic MOF synthesized by the Sun group was also tested for separation of gases (C_1_–C_3_). It was found to compete with other MOFs for acetylene uptake especially [93] and other light hydrocarbons. It takes up to 196.5 cm^3^ g^−1^ at 273 K and 139.5 cm^3^ g^−1^ at 295 K of acetylene [65]. Moreover, it has a good uptake performance for other light hydrocarbons such as C_2_H_4_, C_2_H_6_, C_3_H_6_ and C_3_H_8_ at 273 K and 1 bar, values being 123.1, 137.6, 124.1 and 116.2 cm^3^ g^−1^ respectively (Figure 6). In comparison with other promising MOFs such as UTSA-67a [94] and Cu(etz) [95], UPC-21 has a higher uptake for light hydrocarbons (C_2_–C_3_). Again, it is of great importance to note that UPC-21 displays much higher selectivity for C_2_H_6_/CH_4_ at 295 K (Figure 7) than the previously reported MOFs. The high density of open Cu (II) sites, optimized pore size, and multi-aromatic rings are the main contributions to its advantage over other MOFs in this application area [65].

**Figure 8:**
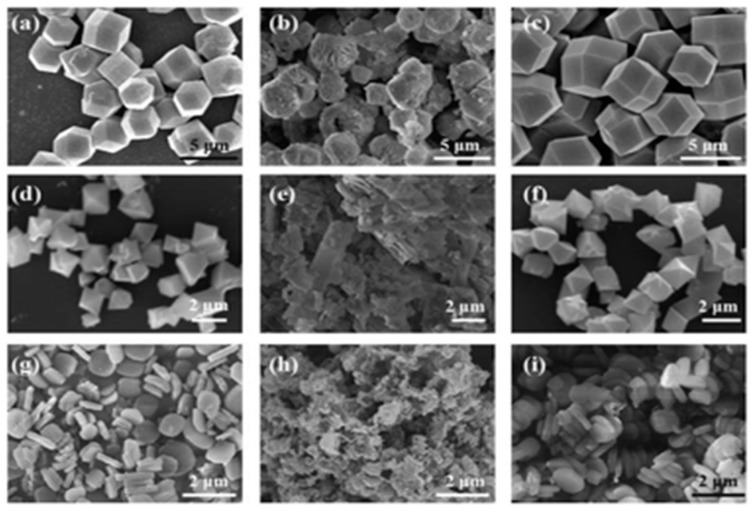
SEM images of (**a**) the AS ZIF-67, (**b**,**c**) the AS ZIF-67 and SH ZIF-67 after exposure to water for 5 days, respectively, (**d**) the AS HKUST-1, (**e**,**f**) the AS HKUST-1 and SH HKUST-1 after exposure to water for 5 days, respectively, (**g**) the AS NH_2_-MIL-125(Ti), (**h**,**i**) the AS NH_2_-MIL-125(Ti) and SH NH_2_-MIL-125(Ti) after exposure to water for 5 days, respectively. Reprinted with permission [66] Copyright 2017, Royal Society of Chemistry.

Considering the capture of CO_2_ from flue gas, a major requirement is that the incoming gas stream is dehydrated since water can bring about a noticeable reduction in the CO_2_ sorption capabilities [96,97,98]. Coating MOF surfaces with a hydrophobic polymer to render them water-resistant was recently illustrated by Qian et al. via a facile solution-immersion process [66]. The approach was attempted on three representative water-sensitive MOFs: NH_2_-MIL-125(Ti), ZIF-67 and HKUST-1 to prove its universality. The MOFs were coated with organosilicon, (DC 1-2577), depositing a layer of hydrophobic high molecular polymer on its external surface, not deeply penetrating the internal pores of the MOFs. The resulting MOFs retained their crystal structure, morphology and surface area when exposed to liquid water, and with a water CA of 146°. The surface hydrophobic MOFs retained up to about 86% of their CO_2_ sorption and desorption capabilities after being exposed to water, while that of the as-synthesized MOFs is about 21%. Scanning Electron Microscope (SEM) images shown in Figure 8 give further proof of the stability of the coated MOFs in water.

Moreover, a group of other scientists with the approach of facile vapor deposition technique, by polydimethylsiloxane (PDMS)-coating treatment [99], made hydrophobic MOFs that were suitable for CO_2_ sorption [29]. PDMS forms a thin protective hydrophobic surface layer on the MOFs, aiding in the retention of the crystalline structures and high porosity under moisture conditions. XRD patterns, BET values and N_2_ sorption isotherms of the coated MOFs remain relatively same after exposure to moisture. However, the original representative water-sensitive MOFs reported otherwise in all cases.

Again, water-stable, zinc-doped Zeolitic Imidazolate Framework-67 (ZIF-67) was synthesized for CO_2_ capture applications [100]. The water stability of ZIF-67 was enhanced by facile Zn-doping during the crystallization process. The MOF exhibited a far better stability in terms of crystal structure, morphology, CO_2_ uptake capacity and surface area as compared to the undoped MOF. This study realized a new approach to improve the water stability of MOFs and a more proper retention ability when exposed to moist environments.

A bridging ligand strategy was used to prepare polyMOFs that were found to be suitable for CO_2_/N_2_ separations [28]. This is because the material reported relatively high CO_2_ sorption but very low N_2_ sorption. The exceptional water stability exhibited by these materials is attributed to the hydrophobicity of polymer ligands and cross-linking of the polymer chains within the MOFs. The following strategies form the bedrock upon which this method was derived. Some earlier reports prepared polymer MOF hybrid materials through PSM, whereby chemical cross-linking of the MOF is achieved through organic ligands to form polymeric monoliths [101,102,103]. Polymerization of polymer chains conducted inside the channels of MOFs has been another approach to synthesizing hybrids of MOFs and polymers [104,105,106].

In addition, a group of researchers has reported a new strategy to divide MOF open channels into confined and hydrophobic compartments by in situ polymerization [107,108,109] of aromatic acetylenes [110]. It was executed by adsorption and encapsulation of 1,2-diethynlybenzene (DEB) monomer into MOF-5 and further heating to a higher temperature to afford polynaphthylene (PN) into the inner channels via Bergman cyclization and subsequent radical polymerization. The resulting MOF, named PN@MOF-5 exhibited a significantly improved moisture stability, doubled CO_2_ capacity (78 vs. 38 cm^3^/g at 273 K and 1 bar) and 23 times higher CO_2_/N_2_ selectivity (212 vs. 9) compared with the pristine MOF-5.

With reference to recent literature, hydrophobic MOFs have been more extensively applied in CO_2_ capture in the broad area of gas separation and storage. Such works have re-visited old ways or invented new strategies of synthesizing hydrophobic MOFs for such a purpose. Among them, creating a permeable hydrophobic layer on the surface of MOFs is the commonest strategy. This strategy has the advantage of avoiding setbacks such as reduced porosity, tedious procedure, complex instrumentation, and so on. Again, intrinsic properties such as surface area, pore texture, and crystalline structure are retained. Using a hydrophobic organic ligand realizes lower surface area compared to the other methods, including the in situ polymerization method. The CO_2_ sorption retention capacities of the hydrophobic MOFs made by the vapor deposition method was much higher (at least 96%) than those of the solution-immersion method (at least 76%), after exposure to moisture. These results may not be solely based on the processes adopted but the ligands and MOFs experimented with as well.

## 4. Other Applications

### 4.1. Self-Cleaning

The function of removing contaminating particles from superhydrophobic surfaces by impacting or rolling water droplets is denoted as self-cleaning [111]. This phenomenon requires external forces such as gravity and is typically referred to as “Lotus Effect” [112,113]. Micro and nanoscale surface roughness, which reduces surface free energy is a prerequisite for such surfaces to exhibit water repellence behavior [114,115,116,117,118]. The guideline for the design of synthetic self-cleaning materials entails size, shape, rigidity and ordering with combined surface micro and nanostructure [119,120,121,122]. Aligned polymer nanofibers [123,124,125], self-assembled monolayer (SAM) modified surfaces [126,127], carbon nanotubes (CNTs) [128,129] and lithographic patterning [130,131,132,133] with very high CAs have been successfully designed. MOFs would definitely present the benefit of inherent porosity and water surface repellence [43,134,135,136,137,138,139,140,141,142,143,144].

**Figure 9:**
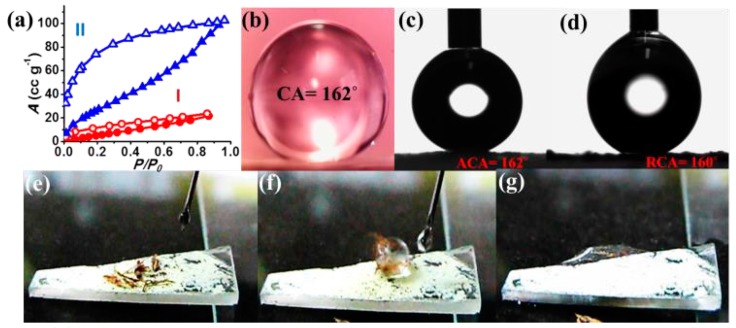
(**a**) Solvent vapor adsorption isotherms of NMOF- at 298 K: I: Water (red) and II: benzene (blue), Po is the saturated vapor pressure, 3.17 kPa (water) and 12.60 kPa (Benzene) at 298 K. Water contact angles of NMOF-1 coated substrate; (**b**) Static contact angle; (**c**) advancing contact angle and (**d**) receding contact angle (**e**–**g**) video snapshots of a self-cleaning experiment showing the removal of dirt from the surface. Reprinted with permission [64] Copyright 2015, Royal Society of Chemistry.

**Figure 10:**
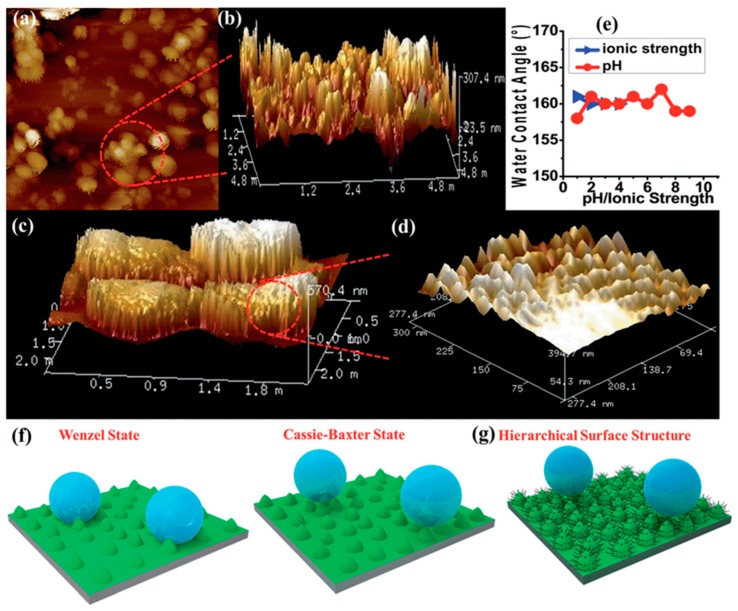
(**a**) 2D-AFM image of NMOF-1 micro-particles on coated glass surface; (**b**) Corresponding 3D-AFM; (**c**) 3D-AFM image of four adjacent microspheres of NMOF-1; (**d**) Corresponding high magnified image showing continuous nano-roughness present on each micro-particle (**e**) Plots showing changes in CA with pH/ionic strength; (**f**) Schematic showing the different states use to explain surface water repellency: left: Wenzel state or the wetting state, right: Cassie-Baxter state or the superhydrophobic state (A transition from the Wenzel to the Cassie-Baxter model occurs when we consider that rough textures on a surface trap air pockets in between); (**g**) Incorporation of hierarchical surface for the generation of the self-cleaning effect in NMOF-1. Reprinted with permission [64] Copyright 2015 Royal Society of Chemistry.

NMOF-1 is a hydrophobic MOF that has been tested for the self-cleaning application [64]. It was synthesized via coordination directed self-assembly of dialkoxyoctadecyl-oligo-(p-phenyleneethynylene) dicarboxylate (OPE-C18) with Zinc II in DMF/H_2_0 mixture. This strategy was adapted from an earlier reported work [27]. OPE-C_18_, being a hydrophobic alkyl chain when exposed on the MOF surface, decreased surface energy hence increasing hydrophobicity. NMOF-1 is thermally stable in that the characteristic PXRD peaks are retained even at temperatures as high as 300 °C. A glass surface was coated with the MOF, dust particles placed on it and water droplets introduced on the surface. The water droplets in no time rolled off the surface laden with the dust particles (Figure 9). This phenomenon depicts the self-cleaning nature of NMOF-1. Field Emission Scanning Electron Microscopy (FESEM) and Atomic Force Microscopy (AFM) jointly confirmed the hierarchal surface roughness nature of NMOF-1, trapped with air pockets (Figure 10). This is the main property of this MOF that contributes to its superhydrophobicity and subsequently its self-cleaning ability. This work realized MOF with a very high CA and corrosion resistance with a pH stability range of 1–9. This makes it a promising material for industrial applications in stain-resistant textiles, anti-biofouling paints in ships and so on.

### 4.2. Liquid Marbles

**Figure 11:**
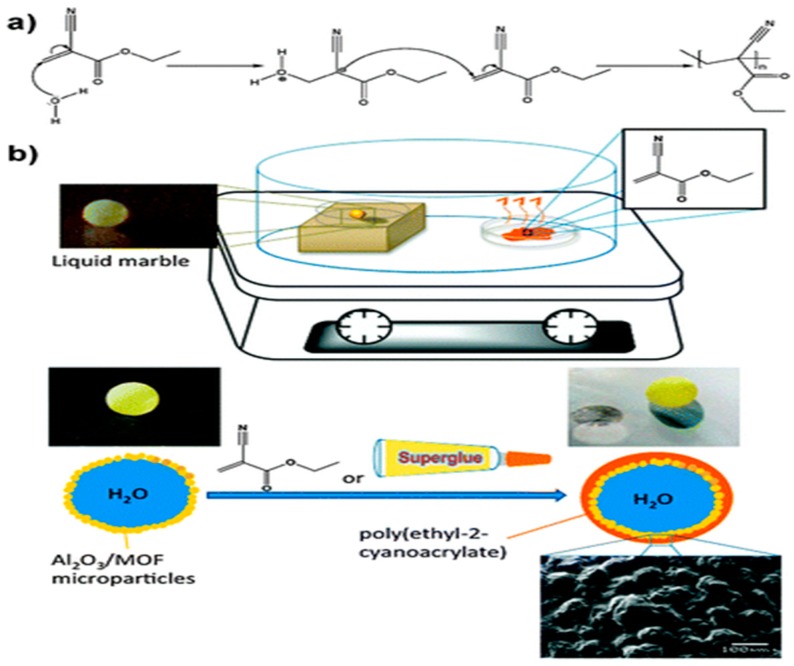
(**a**) Mechanism of cyanoacrylate polymerization; (**b**) Reaction set-up for the interfacial polymerization of ethyl-2-cyanoacrylate on a liquid marble. Heating the hotplate to 80 °C enhances vaporization of the monomer, which condenses on the liquid marble and polymerizes. Reprinted with permission [151] Copyright 2013 Royal Society of Chemistry.

A less common application area for hydrophobic MOFs is liquid marbles. Liquid marble is a ball-like object formed by virtue of a liquid being entirely encapsulated in a hydrophobic powder [145]. Liquid marbles have functioned as miniature reactors [146], micro-pumps [147], pH sensors [148], gas sensors [149] and water pollution sensors [150]. Several hydrophobic particles such as superhydrophobic silica aerogels and Fe_3_O_4_ have been explored in this field but least can be said of hydrophobic MOFs. The perfluorooctyl-modified NH_2_-MIL-53(Al) developed by Chin et al. turned out highly hydrophobic and was used to make liquid marbles [151]. Interfacial polymerization of ethyl-2-cyanoacrylate was further carried out on the outer surface of the marbles to produce stable liquid capsules (Figure 11). This work revealed that this approach to interfacial polymerization can be used industrially for the economical formation of biodegradable liquid capsules.

Liquid marbles have broadly served as micro-reactors for polymerization [152] and other reactions [146]. Their property of low adhesion to surfaces makes them act well as non-wetting liquid droplets that display various dynamic characteristics; liquid droplets can be manipulated. MOFs being materials with several advantages can make a significant contribution to the liquid marbles story.

## 5. Summary and Outlook

This review has given a concise update on the recent applications of hydrophobic MOFs in general. The research on hydrophobic MOFs began about a decade ago and just like any other material explored in science, they come with their imperfections. The evolution of this special kind of MOFs came about as scientists attempted to solve the chemical and thermal instability nature of most MOFs, as they were known to be mostly moisture-instable. Scientists managed to solve this particular problem and obviously produced new application areas for MOFs, which is a very recommendable achievement. However, scientists should produce water-stable MOFs that are still applicable to the removal of contaminants in wastewater other than oils as this is the main advantage activated carbon has over MOFs.

One major application area of hydrophobic MOFs is in oil-water separation and most of these MOFs can do just that. Researchers should then move to the next step of developing hydrophobic MOFs that can further separate these oils into the various classes of hydrocarbons, which is a more successful recovery of oils. We think that designing MOFs with specific pore sizes would be a stepping stone to overcoming this problem. The advantage is that for their practical use, these hydrocarbons are required at high quality and purity.

Furthermore, the application areas of hydrophobic MOFs as it stands now are limited; an area such as catalysis, if explored, will bring about an unrecoverable revolution in hydrophobic MOFs. In an area such as the Fischer-Tropsch Synthesis, it is thought that hydrophobicity can partially suppress water-gas shift reaction and ultimately decrease carbon dioxide production; hydrophobic MOFs could be of use here. Again, we recommend researchers to study the areas of liquid marbles and self-cleaning more. Moreover, it is recommended that researchers look at applying hydrophobic MOFs whose applications are yet to be explored. This is likely to reveal some major setbacks in designing and synthesizing hydrophobic MOFs, bringing about more novel methods of producing hydrophobic MOFs that have lesser and lesser disadvantages in the various application fields.

MOFs find themselves in a vast area of application in physics, biology, chemistry, engineering and so on. The untiring collaboration of scientists from a plethora of fields will achieve endless solutions to problems in the society.

## Figures and Tables

**Table 1:** Properties of hydrophobic MOFs highlighting contact angle (CA), postsynthetic modification (PSM), SC (self-cleaning), pH stability range (SR), moisture stability (MS), thermal stability, ionic solution stability range (ISSR), Brunauer–Emmett–Teller (BET) surface area (SA) and n-octadecylphosphonic acid (OPA).

MOF	CA (°)	PSM	SC	pH SR	MS	TS	ISSR	BET SA (m^2^·g^−1^)	References
SH ZIF-90	152.4	Yes	-	-	Yes	Yes	-	1182	[63]
OPA-UiO-66	160	Yes	-	1–11	Yes	-	1–2	1068	[62]
OPA-UiO-66-SO_3_H	˃150	Yes	-	1–11	Yes	-	1–2	1148	[62]
OPA-PCN-222	˃150	Yes	-	1–11	Yes	-	1–2	1618	[62]
UHMOF-100	176	No	-	-	Yes	Yes	-	469.2	[32]
NMOF-1	162	No	Yes	1–9	Yes	Yes	1–4	-	[64]
UPC-21	≥145	No	-	-	Yes	-	-	1725.1	[65]
SH NH_2_-MIL-125(Ti)	≥146	Yes	-	-	Yes	-	-	≈1134	[66]
SH ZIF-67	≥146	Yes	-	-	Yes	-	-	≈1587	[66]
SH HKUST-1	≥146	Yes	-	-	Yes	-	-	≈1386	[66]
